# Morphological Characterization, Interaction Mechanisms, and Functional Properties of a Non-Covalent Whey Protein Isolate–Ellagic Acid Complex

**DOI:** 10.3390/foods15152689

**Published:** 2026-07-30

**Authors:** Lingtong Fan, Juexi Liu, Yan Yang, Qingsong Liu, Danjun Guo, Ouyan Han, Wei Xu, E Liao, Huajuan Wang

**Affiliations:** 1 College of Food Science and Engineering, Wuhan Polytechnic University, Wuhan 430023, China; fanlt2005@163.com (L.F.); ljx4169161107@163.com (J.L.); 13006336981@163.com (Y.Y.); lqs2892013390@163.com (Q.L.); xuwei1216@163.com (W.X.); liaoe1987@whpu.edu.cn (E.L.); wanghuajuan0609@126.com (H.W.); 2 Hubei Key Laboratory for Processing and Transformation of Agricultural Products, Wuhan 430023, China

**Keywords:** whey protein isolate (WPI), ellagic acid (EA), non-covalent interaction, sarcopenia

## Abstract

Sarcopenia, characterized by loss of muscle mass and strength, causes difficulty in standing and walking and increases fracture risk in older adults, making its prevention a priority for healthy aging. Whey protein isolate (WPI) promotes muscle protein synthesis, while ellagic acid (EA), a polyphenol, alleviates symptoms by reducing oxidative stress. However, WPI is prone to oxidative damage during processing, and EA suffers from low stability and bioaccessibility. For these reasons, a non-covalent complex was prepared from WPI and EA, and its preparation conditions were systematically optimized through single-factor experiments followed by orthogonal design. The objectives were to enhance the stability and bioaccessibility of EA through the protective effect of WPI, thereby enabling synergistic anti-sarcopenia effects. Multi-spectroscopic techniques and molecular simulations were employed for morphological characterization and interaction analysis. The optimal preparation conditions were pH 5.0, a 2 h reaction, and a WPI: EA molar ratio of 1:2.5. Under these conditions, the antioxidant activity of the WPI-EA non-covalent complex increased by 27.20% (*p* < 0.05). At the same time, protein digestibility decreased by 3.04% (*p* < 0.05). EA bound non-covalently near the tryptophan and tyrosine residues of WPI, altering its secondary and tertiary structures. WPI-EA non-covalent complex exhibited a 38.94% reduction in its surface hydrophobicity (*p* < 0.05) and a 2.42% increase in α-helix content (*p* < 0.05). These conformational changes provided a structural basis for the improved bioaccessibility of WPI. The spectroscopic observations were corroborated by molecular docking and MD simulations, which revealed that both hydrophobic interactions and hydrogen bonds contributed to the stable binding of EA to β-Lg, with hydrophobic contacts predominating in the binding mode and hydrogen bonds playing a critical role in maintaining conformational stability throughout the simulation. In summary, the WPI-EA non-covalent complex exhibited good antioxidant activity, indicating its potential as a functional ingredient for sarcopenia management and for improving skeletal muscle health in aging individuals.

## 1. Introduction

Sarcopenia has become a significant public health issue with the rapid global population aging, marked by progressive losses in muscle mass, strength, and physical performance. This condition undermines the independence of older adults, increases the likelihood of falls, fractures, and mortality, and consequently imposes a substantial strain on healthcare systems [1,2]. Inadequate protein intake and anabolic resistance are core contributors. Older adults have lower sensitivity to leucine and require more high-quality protein to sustain protein synthesis [3]. Whey protein isolate (WPI), rich in protein, highly digestible, and abundant in branched-chain amino acids, effectively boosts muscle protein accretion and alleviates oxidative stress in the elderly, making it a preferred choice for nutritional intervention [4]. McKendry et al. confirmed that supplemental WPI significantly increased myofibrillar protein synthesis rate and improved mTORC1 phosphorylation in healthy older men [4]. Additionally, the anti-sarcopenic potential of whey protein isolate has been reported in recent studies. Li et al. [5] found that daily intake of 9.6 to 40 g of whey protein significantly increased appendicular skeletal muscle mass index and muscle mass in sarcopenic elderly individuals. Moreover, whey protein supplementation combined with resistance training was more effective than resistance training alone in improving grip strength and skeletal muscle mass [6]. However, oxidizing enzymes and unsaturated fatty acids in WPI raw materials can induce oxidative deterioration, thereby reducing their nutritional quality [7].

Polyphenolic compounds also possess anti-sarcopenic potential. Polyphenols, as secondary metabolites in fruits, vegetables, and tea, exhibit strong antioxidant activity due to their phenolic hydroxyl groups [8,9,10]. Evidence indicates that polyphenols act on the mTOR, NF-κB, and AMPK signaling pathways, leading to the mitigation of oxidative stress and chronic inflammation, improvement of mitochondrial energy metabolism, and maintenance of skeletal muscle mass [8]. Among polyphenols, ellagic acid (EA) has attracted particular interest for its potential muscle-protective effects. Li et al. [11] demonstrated that dietary EA at 75 and 150 mg/kg increased slow myosin heavy chain protein, slow-twitch fiber number, and malate dehydrogenase activity in the longissimus thoracis muscle of pigs, while decreasing fast myosin heavy chain and fast-twitch fibers. In porcine satellite cells, EA at 10 μmol/L elevated mitochondrial DNA content, upregulated ATP5G and TFAM mRNA expression, and enhanced AMPK phosphorylation [11]. Collectively, these findings suggest that EA modulates muscle fiber composition and promotes mitochondrial biogenesis through AMPK signaling. Furthermore, EA attenuates muscle inflammation by modulating the Nrf-2/NF-κB signaling pathway and suppressing pro-inflammatory cytokine expression [12,13]. Nevertheless, the application of these compounds in functional foods is constrained by inadequate chemical stability and limited bioavailability [14].

Notably, proteins readily form complexes with dietary polyphenols during digestion. Yan et al. [15] reported that the WPI-EGCG complex improved the bioavailability, UV stability, and processability of EGCG, while also enhancing the formability and emulsifying capacity of WPI. Mao et al. [14] showed that after non-covalent binding of blackcurrant polyphenols to milk proteins, polyphenol bioaccessibility and antioxidant activity were significantly higher than those of free polyphenols. The interactions between proteins and polyphenols fall predominantly into two categories: non-covalent and covalent. Non-covalent interactions are mild and reversible, dominated by hydrophobic forces, with hydrogen-bonding and electrostatic contributions, thereby improving protein functionality without affecting bioactivity [16]. Covalent interactions are irreversible, often requiring high temperature, enzymes, or alkaline conditions; although they can enhance stability [17], they may reduce protein digestibility and nutritional value [18]. In contrast, non-covalent interactions avoid these drawbacks and therefore offer greater application advantages. The studies discussed above indicate that the construction of protein-polyphenol complexes through non-covalent interactions can simultaneously enhance the functional properties of proteins and improve the stability of polyphenols under mild conditions. This strategy offers a feasible solution to the challenges of WPI susceptibility to oxidation and the limited bioaccessibility of EA.

Despite extensive research on the non-covalent interactions between WPI and various polyphenols, most studies have focused on improving techno-functional properties such as emulsification, foaming, and solubility, while relatively little attention has been paid to the bioactivities of the resulting complexes. In addition, EA, a polyphenol containing four phenolic hydroxyl groups and two lactone rings, has not been systematically investigated for non-covalent complexation with WPI. Therefore, in this study, a WPI-EA non-covalent complex was constructed to enhance the antioxidant capacity of WPI and the stability of EA. The fabrication parameters were optimized through single-factor trials and orthogonal experiments, and the complex was characterized by multiple spectroscopic techniques and scanning electron microscopy. Furthermore, molecular docking and molecular dynamics simulations were performed to elucidate the conformational characteristics, association modes, and interaction sites at both the molecular and computational levels. Overall, this work provides a theoretical and material foundation for future functional evaluation of the WPI-EA non-covalent complex in sarcopenia-related research.

## 2. Materials and Methods

### 2.1. Materials

WPI (purity 90%), ellagic acid (purity 90%), and dialysis bags (3500 Da) were purchased from Shanghai Yuanye Biotechnology Co., Ltd. (Shanghai, China). NaH_2_PO_4_, Na_2_HPO_4_, NaOH, HCl, and DMSO were purchased from Sinopharm Chemical Reagent Co., Ltd. (Shanghai, China). The Bradford Protein Concentration Assay Kit was purchased from Biosharp Co., Ltd. (Beijing, China). SGF, SIF, and 1,1-diphenyl-2-picrylhydrazyl (DPPH) were purchased from Beijing Regen Biotechnology Co., Ltd. (Beijing, China). 8-anilino-1-naphthalenesulfonic acid (ANS) was purchased from Shanghai Aladdin Biochemical Technology Co., Ltd. (Shanghai, China).

### 2.2. Preparation and Process Optimization of WPI-EA Non-Covalent Complex

The preparation of the WPI-EA non-covalent complex was carried out according to the method of Ferraro et al. [19] with modifications to optimize the complexation conditions. The molar concentration of WPI was calculated based on its predominant protein component, β-lactoglobulin (β-Lg, molecular weight 18,400 g/mol), which constitutes approximately 70% of WPI and serves as the primary protein fraction involved in polyphenol binding, while EA molarity was based on its molecular weight (302.2 g/mol), following the widely adopted approach in protein-polyphenol interaction studies [19,20]. WPI powder (0.92 g) was dissolved in 0.1 M PBS (pH 7.0) to a final volume of 100 mL, yielding a 500 μM (9.2 mg/mL) solution. The mixture was stirred at 400 rpm and 25 °C for 2 h to achieve full dissolution and then refrigerated at 4 °C overnight. Separately, a 100 mM EA stock solution in DMSO was prepared and then diluted with PBS to the appropriate concentrations. At 25 °C, the WPI concentration was fixed at 500 μM, and the EA working solutions at different molar ratios were prepared accordingly. For each EA working solution, an equal volume of the WPI solution was added, and the resulting mixture was stirred at 400 rpm for 2 h under light-protected conditions to allow the reaction to proceed. To ensure efficient binding and prevent protein aggregation, the reaction temperature was maintained at 25 °C throughout the complexation process. The reaction mixture was then transferred into a dialysis bag and dialyzed against PBS (pH 7.0) at 4 °C for 48 h to remove unbound EA monomers, with the external buffer renewed every 6–8 h, and the pH was maintained by adding 1 M NaOH or HCl as required until the absorbance at 300 nm of the dialysate returned to a value close to the baseline. Lyophilization of the dialysate for 48 h afforded the WPI-EA non-covalent complex in powder form. The effects of pH (4.5, 5.0, 5.5, 6.0, 6.5), WPI: EA molar ratio (1:0.1, 1:0.5, 1:1, 1:2, 1:4), and reaction time (1.0, 1.5, 2.0, 2.5, 3.0 h) on both the DPPH radical-scavenging activity and protein digestibility of the complexes were assessed through single-factor trials, to identify the most favorable preparation parameters.

Using DPPH scavenging and protein digestibility as indicators, the effects of pH, reaction time, and reactant molar ratio on the complexes were examined using single-factor data. The preparation method for the non-covalent complex was optimized using an L_9_(3^4^) orthogonal test, with the experimental factors and levels listed in Table S1. The orthogonal test employed a comprehensive scoring scheme based on intuitive analysis, which gave DPPH radical scavenging rate and protein digestibility weighting factors of 0.6 and 0.4, respectively. To determine the comprehensive score, we first calculated the degree of affiliation for each indicator. The formulae for both the affiliation degrees and the comprehensive score are given below:

(1) $$Affiliation=\frac{Value-Minimum}{Maximum-Minimum},$$

(2) $$\begin{array}{c} Comprehensive\;score=Affiliation\;of\;DPPH\;clearance\times 0.6\\ +Affiliation\;of\;protein\;digestibility\times 0.4, \end{array}$$

A verification experiment was conducted under the optimal conditions determined by the orthogonal test. Following *in vitro* digestion, the complex was evaluated for its anti-sarcopenic potential by assessing DPPH scavenging activity, protein digestibility, and essential amino acid concentrations.

### 2.3. Measurement of DPPH Radical Scavenging Rate

Following the method of Liang et al. [21], a 1 mM DPPH solution was prepared in absolute ethanol and stored at 4 °C in the dark; it was diluted 10-fold before use. Then, three separate mixtures were prepared, each containing 3.0 mL of 0.1 mM DPPH in ethanol: one with 3.0 mL of distilled water (*A*_0_), another with 3.0 mL of sample solution at 1 mg/mL (*A_i_*), and a third with 3.0 mL of absolute ethanol (*A_j_*). These were allowed to react for 30 min at ambient temperature in the dark, after which the absorbance at 517 nm was measured. All tests were run in triplicate. DPPH scavenging activity was determined using the formula given below:

(3) $$DPPH\;scavenging\;activity\left(\%\right)=\left[1-\frac{A_{i}-A_{j}}{A_{0}}\right]\times 100,$$

*A*_0_: Absorbance at 517 nm of the mixture containing distilled water and DPPH in ethanol.
*A_i_*: Absorbance at 517 nm of the mixture containing the sample solution and DPPH in ethanol.
*A_j_*: Absorbance at 517 nm of the mixture containing the sample solution and ethanol alone.

### 2.4. Measurement of In Vitro Digestibility of WPI-EA Non-Covalent Complex

*In vitro* digestion was performed using a model adapted from Liu et al. [22] and in reference to the INFOGEST standardized static digestion protocol [23]. A 5 mg/mL solution of the complex was prepared using 0.01 M phosphate buffer (pH 7.0), with the buffer alone serving as the blank. To mimic gastric conditions, 5 mL of each test solution (sample or blank) was combined with an equal amount of gastric simulant (pH 1.2) containing porcine pepsin (2000 U/mL, final activity), 2 g/L NaCl, 0.32 g/L KCl, 0.12 g/L CaCl_2_·2H_2_O, 0.05 g/L KH_2_PO_4_, and 0.05 g/L MgCl_2_·6H_2_O. The mixture was agitated at 100 rpm for 2 h at 37 °C. Thereafter, the digest was cooled in an ice bath, neutralized to pH 6.8–7.0 to halt digestion, and then combined with 10 mL of intestinal simulant (pH 6.8) containing pancreatin (100 U/mL, final activity based on trypsin activity), 6.8 g/L KH_2_PO_4_, 0.62 g/L KCl, 0.11 g/L CaCl_2_·2H_2_O, and 0.05 g/L MgCl_2_·6H_2_O. To mimic the intestinal phase, the mixture was kept at 37 °C under agitation for 2 h, followed by a 10 min boiling step to stop enzymatic activity. Following digestion, the sample was centrifuged at 10,000 rpm at 4 °C for 10 min, and the resulting supernatant was collected for further use. Protein concentrations in the pre-digestion complex solution and in the post-digestion supernatant were quantified using the Bradford method. Digestibility was then calculated using the following equation:

(4) $$Protein\;digestibility=\frac{m_{2}-m_{0}}{m_{1}}\times 100,$$

m_0_: Protein concentration in the supernatant of the blank group following the digestion process.
m_1_: Protein concentration of the complex before digestion.
m_2_: Protein concentration in the supernatant obtained from the digested sample.

### 2.5. Measurement of Amino Acid Content

*In vitro* digestion of the WPI-EA non-covalent complex was carried out in accordance with the procedure described in Section 2.4. The digest was transferred into a 500-Da dialysis bag and immersed in distilled water at 25 °C under magnetic stirring (400 rpm) for 2 h. Subsequently, the outer liquid was collected, concentrated by rotary evaporation, and lyophilized. The amino acid levels were determined according to the GB 5009.124-2016 method [24]. The freeze-dried sample (about 0.3500 g) was placed in a digestion tube, to which 10 mL of 6 M HCl and 3–4 drops of phenol were added. The tube was then dipped in liquid nitrogen for 3 min, after which it was evacuated and filled with nitrogen before sealing. The sealed tube was hydrolyzed at 110 ± 1 °C in a forced-air oven for 22 h. Upon completion of hydrolysis, the resulting liquid was filtered, and the filtrate was subsequently diluted to a final volume of 50 mL. The filtrate was then passed through a 0.22 μm membrane and subsequently analyzed for its amino acid concentration.

### 2.6. Ultraviolet (UV)–Visible Spectroscopy Measurement

WPI-EA non-covalent complex and WPI solutions were prepared at concentrations of 0.1, 0.5, and 1.0 mg/mL according to the protocol described by Ma et al. [25]. With distilled water as the reference, spectral data were acquired over 190–750 nm at a 0.1 nm resolution using a Thermo Fisher Scientific spectrophotometer (Waltham, MA, USA), and each sample was scanned three times. Baseline correction was performed using the distilled water blank; no smoothing was applied to the raw spectra to preserve spectral features.

### 2.7. FT-IR Spectroscopy Measurement

The method of Liu et al. [26] was followed to analyze the secondary structure of the WPI-EA non-covalent complex using an IR Prestige-21 Fourier transform infrared spectrometer (Shimadzu, Kyoto, Japan). Lyophilized samples with WPI concentrations of 3.68, 9.20, and 14.72 mg/mL were each mixed with KBr at a 1:100 ratio (g/g), ground, and pressed into transparent disks. Spectral data were recorded over the range of 400–4000 cm^−1^ at a resolution of 4 cm^−1^ with 32 scans. Baseline correction and smoothing were performed using the automatic functions in OMNIC 9.7 software. Peak detection was performed using the peak analysis module of Origin 2022. Subsequently, the amide I band (1600–1700 cm^−1^) was deconvoluted and curve-fitted using Peakfit 4.12 to calculate the relative proportions of protein secondary structures.

### 2.8. Intrinsic Fluorescence Spectroscopy Measurement

Referring to the method of Hellwig [27], WPI-EA non-covalent complex and WPI solutions at 0.05, 0.1, and 0.2 mg/mL were placed in quartz cuvettes. Emission spectra were acquired on a F-4500 spectrophotometer (HITACHI, Tokyo, Japan) from 300 to 450 nm with an 8.0 s response time, using a 280 nm excitation wavelength, a 5.0 nm slit, a 1200 nm/min scanning rate, and a 750 V voltage. Three replicate scans were performed for each sample, and the average was taken. A blank control prepared with distilled water was used for baseline correction, and the acquired spectral data were automatically smoothed using Origin 2022 software.

### 2.9. Synchronous Fluorescence Spectroscopy Measurement

The method of Sui et al. was followed [28]. Solutions of WPI and WPI-EA non-covalent complexes (0.05, 0.1, and 0.2 mg/mL) were transferred to quartz cuvettes for synchronous fluorescence measurements of tyrosine (Δλ = 15 nm) and tryptophan (Δλ = 60 nm). Spectra were collected from 200 to 350 nm, with emission maxima set at 215 nm and 260 nm, at a scan rate of 1200 nm/min, a 5.0 nm slit, 700 V, and a 0.50 s response. Each measurement was repeated in triplicate, and the results were averaged. Distilled water was used as the blank for baseline correction, and the spectra were subsequently smoothed using Origin 2022 software.

### 2.10. Three-Dimensional Fluorescence Spectroscopy Measurement

Referring to the protocol of Allahdad et al. [29], ultrapure water served as the blank. WPI-EA non-covalent complex and WPI solutions at 0.05 mg/mL were placed in quartz cuvettes. Using the 3D-scan function, fluorescence excitation was scanned over 200–350 nm and emission over 200–500 nm, with instrumental settings of 1200 nm/min, 2 nm step size, 5.0 nm slit width, and 700 V. Rayleigh scattering was removed using MATLAB R2025a to eliminate scattered-light interference.

### 2.11. Surface Hydrophobicity (H_0_)

Surface hydrophobicity (H_0_) was evaluated according to the method described by Meng et al. [30], using 8-anilino-1-naphthalenesulfonic acid (ANS) as the fluorescent probe. A 20 µL portion of 8 mM ANS was combined with 4 mL of WPI or WPI-EA non-covalent complex solutions (protein content 0.0125–0.1 mg/mL) and incubated in the dark for 15 min. Using a F-4500 fluorometer (HITACHI, Tokyo, Japan) (390 nm excitation and 470 nm emission, 5.0 nm slit, 500 V), fluorescence intensity was measured in distilled water containing ANS, with the blank used for background subtraction. H_0_ was derived from the initial slope of intensity versus protein concentration.

### 2.12. Microstructure of the Complex

According to Zhong et al. [31], approximately 1 mg of each sample (WPI-EA non-covalent complex and WPI powder) was placed in a sample holder using double-sided conductive tape, coated with a gold layer, and finally observed under a JMS-6700F SEM (JEOL, Tokyo, Japan) with an acceleration voltage of 15 kV. Morphological images were captured at magnifications of 500× and 1500×, respectively.

### 2.13. Molecular Docking

As β-lactoglobulin (β-Lg) accounts for over 70% of WPI, it was selected as the model protein to investigate the interaction between EA and WPI. EA was retrieved from the PubChem database (https://pubchem.ncbi.nlm.nih.gov/) (accessed on 5 February 2026) under CID 5281855. Its 3D structure was prepared, and coordinates were assigned using the Ligprep module in Schrödinger with the OPLS_4 force field, while possible stereoisomers and protonation states were generated with the Epik module. The 3D coordinates of β-Lg (PDB entry 3NPO, resolution 2.20 Å) were obtained from the RCSB PDB website (https://www.rcsb.org/) (accessed on 5 February 2026). Protein preparation was performed with the Protein Preparation Wizard, which included bond order assignment, hydrogen addition, removal of water and cofactors, optimization of the hydrogen-bond network, and energy minimization under the OPLS_4 force field. Docking was carried out using the Glide module, with binding pockets predicted by SiteMap. The outer docking box was set to the dimensions of the predicted site, the inner box to 10 Å, and the center to the site centroid. Docking was run in standard precision (SP) mode, using the docking score as the scoring function. For each simulation, ten ligand conformations were generated and subjected to post-docking minimization; the conformation with the highest docking score was then selected for further analysis.

### 2.14. Molecular Dynamics Simulation

Following the method of Abraham et al. [32], the best docking pose of EA with β-Lg was taken as the initial MD structure. PROPKA 3.0 was employed to assign protonation states to protein residues, while all crystallographic water molecules originally present were preserved. The System Builder in Schrödinger was used to construct the all-atom complex, which was then parameterized with OPLS_4, solvated with TIP3P water, and surrounded by a 10.0 Å buffer layer. The system was first subjected to energy minimization for 50,000 steps using the steepest descent algorithm. Subsequently, NVT and NPT equilibrations were performed for 50,000 steps each at 300 K and 1 atm, with positional restraints applied to heavy atoms. After equilibration, a 100 ns unrestrained MD simulation was conducted, with frames captured every 10 ps. Schrödinger’s 2023 analysis provided RMSD, RMSF, Rg, secondary structure, interactions, and MM-GBSA binding free energy.

### 2.15. Statistical Analysis of Data

Fluorescence spectral data were recorded and analyzed with Hitachi FL Solutions 2.1. All assays were carried out in triplicate using three independently prepared sample batches (biological replicates, *n* = 3). All values were expressed as means ± SD from the three biological replicates. The homogeneity of variances was verified using Levene’s test. Data visualization was performed using Origin 2022 and GraphPad Prism 8.0, while statistical comparisons were conducted with Duncan’s multiple range test in SPSS 26.0 (*p* < 0.05). Exact *p*-values are reported in the relevant tables in the Supplementary Materials. 95% confidence intervals are provided for key parameters where applicable.

## 3. Results and Discussion

### 3.1. Construction of WPI-EA Non-Covalent Complex and Optimization of the Process

**Figure 1:**
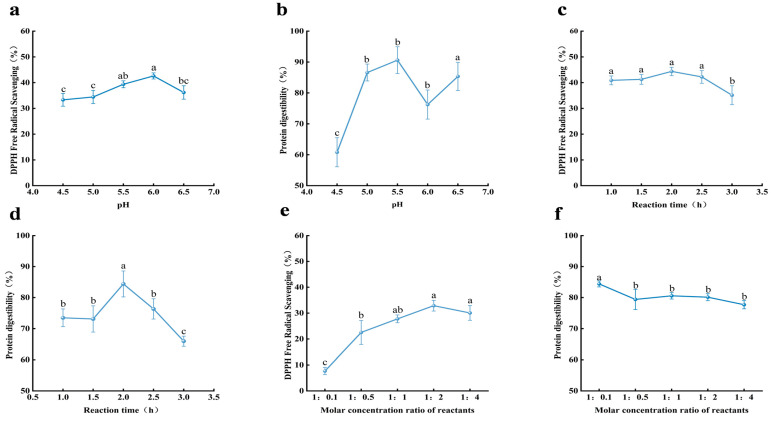
DPPH radical scavenging and *in vitro* protein digestibility of the WPI-EA non-covalent complex were assessed under varying pH (**a**,**b**), reaction time (**c**,**d**), and reactant concentration (**e**,**f**). Note: Statistically significant differences are indicated by distinct superscript letters (*p* < 0.05), *n* = 3.

Figure 1a,b shows that the WPI-EA non-covalent complex reached its maximum protein digestibility (86.61 ± 2.73%) at pH 5.5. In comparison, relatively high DPPH scavenging capacity (39.37 ± 1.35%) was also observed under the same conditions. The pH can regulate protein surface charge and conformation, thereby affecting the accessibility of polyphenol binding sites. Under weakly acidic conditions, the WPI structure is relatively loose, which facilitates non-covalent binding to polyphenols and the expression of their functional properties [20]. Thus, pH 5.5 was selected. As shown in Figure 1c,d, at a 2 h reaction time, the DPPH radical scavenging activity of the WPI-EA non-covalent complex reached 44.37 ± 1.62%, and the protein digestibility was 84.39 ± 4.18%. Non-covalent binding typically reached equilibrium within a relatively short time, whereas prolonged reaction times may lead to partial complex aggregation [30]. Hence, 2 h was chosen. Figure 1e,f shows that the maximum DPPH scavenging capacity (32.87 ± 2.08%) occurred at a 1:2 molar ratio of WPI to EA, and the digestibility was also fairly high (80.12 ± 1.12%) under this condition. Li et al. [33] demonstrated that the binding ratio between polyphenols and whey protein significantly influences the functional characteristics of the resulting complex and that an appropriate ratio enhances the antioxidant activity of polyphenols. Therefore, a 1:2 ratio was used.

Homogeneity of variances was assessed before one-way ANOVA. Levene’s test confirmed homogeneity of variances across all groups for DPPH scavenging activity (pH: *p* = 0.251; reaction time: *p* = 0.297; molar ratio: *p* = 0.271) and for protein digestibility (pH: *p* = 0.862; reaction time: *p* = 0.299; molar ratio: *p* = 0.439), thus meeting the assumptions for parametric analysis. The subsequent ANOVA revealed significant effects of all three factors on both DPPH scavenging activity and protein digestibility. Detailed ANOVA results are provided in Tables S4 and S5.

From Tables S2 and S3, the comprehensive scores for DPPH scavenging and protein digestibility of the WPI-EA non-covalent complex showed that the factors ranked as reaction time > pH > reactant molar ratio. The optimized fabrication parameters were determined to be pH 5.0, a WPI-to-EA molar ratio of 1:2.5, and a reaction period of 2 h. Under these conditions, the DPPH scavenging activity and protein digestibility reached 48.10 ± 0.20% (*p* < 0.05) and 87.42 ± 4.09% (*p* < 0.05), respectively.

### 3.2. Determination of Amino Acid Profile in the Digestate of the WPI-EA Non-Covalent Complex

Table 1 presents the post-digestion BCAA levels for WPI and the WPI-EA non-covalent complex, which were 22.97% and 21.77%, respectively. WPI is rich in BCAAs, which are known to promote muscle protein synthesis and skeletal muscle development by activating the mTORC1 signaling pathway [34]. The total contents of essential amino acids were 31.18% and 31.02%, and both samples showed no marked variation in BCAA or EAA levels, consistent with the FAO/WHO reference pattern. Hydrophobic amino acid contents were 37.49% and 36.86%, and glycine contents were 1.80% and 2.05%, respectively, with no notable changes observed. Hydrophobic amino acids can donate hydrogen atoms via their indole or benzene rings, thereby inhibiting lipid peroxidation, boosting antioxidant capacity, and reducing myocyte apoptosis [35]. Glycine attenuates age-related oxidative stress and muscle protein breakdown [36,37]. However, the total amino acid content of the WPI-EA non-covalent complex after *in vitro* digestion (9.74 g/100 g) was 20.10% lower than that of WPI (12.19 g/100 g). A similar observation was reported by Budryn et al. [38], which may be attributable to EA binding that covers protease cleavage sites or alters protein conformation, thereby reducing enzymatic susceptibility. More notably, cysteine content dropped from 0.63 to 0.22 g/100 g, a decrease of 65.08%. This marked reduction may arise from the greater susceptibility of cysteine to oxidation due to its lower tendency to form disulfide bonds in the presence of polyphenols [39] or from the nucleophilic addition of cysteine thiols to EA or its oxidized quinone derivatives [40]. Overall, the amino acid analysis revealed that although total amino acids and cysteine decreased, the key functional amino acids (BCAAs, EAAs, hydrophobic amino acids, and glycine) were largely preserved. This preservation was consistent with the increased DPPH scavenging activity and enhanced antioxidant capacity of the WPI-EA non-covalent complex compared to WPI, as well as with its maintained high protein digestibility. These results confirm the potential of the WPI-EA non-covalent complex as a functional component for sarcopenia management.

### 3.3. Ultraviolet (UV)–Visible Spectroscopy Analysis

**Figure 2:**
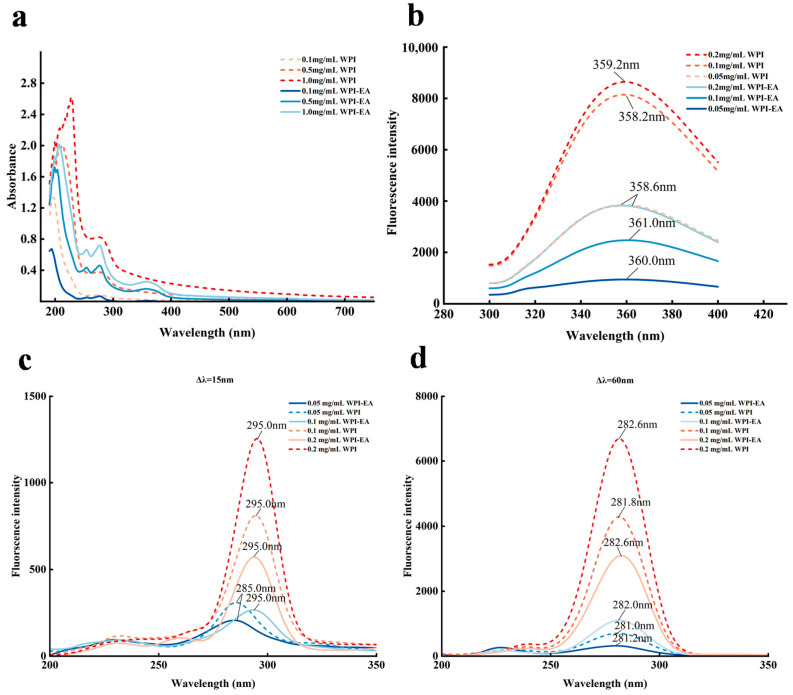
Ultraviolet–visible spectra of 0.1, 0.5, and 1.0 mg/mL WPI and WPI-EA non-covalent complex (**a**). Intrinsic fluorescence of 0.05, 0.1, and 0.2 mg/mL WPI and WPI-EA non-covalent complex (**b**). Synchronous fluorescence of (0.05, 0.1, and 0.2 mg/mL) WPI and WPI-EA non-covalent complexes at Δλ = 15 nm (**c**) and Δλ = 60 nm (**d**).

From Figure 2a, the absorption peaks of WPI at protein concentrations of 0.1, 0.5, and 1.0 mg/mL were at 198, 211, and 227 nm, while WPI-EA non-covalent complex peaks blue-shifted to 194, 199, and 206 nm, accompanied by a distinct hypochromic effect. These absorption bands primarily arose from the n→π* transition of the peptide-bond C=O groups [41]. The blue shift and hypochromism suggested EA-induced folding of the WPI peptide chain and an altered chemical microenvironment around peptide bonds [42]. Notably, the blue shift decreased as WPI concentration increased, suggesting that protein aggregation at higher concentrations may affect EA binding to the peptide backbone. In addition, a weak absorption peak was observed at 276 nm for all WPI samples, whereas the corresponding peak of the WPI-EA non-covalent complex appeared at 275 nm, also with hypochromism. This peak was primarily attributed to aromatic amino acid residues such as tryptophan and tyrosine; its decreased intensity indicated that EA binding changed the microenvironment around these residues [33]. Collectively, the blue shift in the peptide bond absorption in the short-wavelength region, together with the slight spectral shift and hypochromism of the aromatic residue absorption near 275 nm, demonstrated that EA induced WPI peptide chain folding, with aromatic hydrophobic groups buried inward and intramolecular hydrophobic interactions enhanced.

### 3.4. Intrinsic Fluorescence Spectroscopy Analysis

Upon excitation at 280 nm, WPI exhibited intrinsic emission derived from its aromatic amino acid components, including tryptophan and tyrosine. The fluorescence spectra reflected changes in tertiary structure and residue microenvironment. From Figure 2b, the fluorescence emission peaks of WPI at 0.05, 0.1, and 0.2 mg/mL were all located at 358–359 nm, indicating that tryptophan residues primarily contributed to the fluorescence signal. Compared to WPI, the fluorescence emission peaks of the WPI-EA non-covalent complex at 0.05 and 0.1 mg/mL exhibited red shifts of 1.4 nm and 2.8 nm, respectively, whereas a 0.6 nm blue shift was observed at 0.2 mg/mL. The inner filter effect may explain the reversed trend observed at the highest tested concentration [43]. After excluding this interference, the red shifts at low concentrations more reliably reflect the conformational changes induced by EA binding. The observed red shift indicated greater polarity and reduced hydrophobicity in the vicinity of tryptophan, suggesting that EA induced extension of the WPI peptide chain and loosening of the tertiary structure. This is consistent with reports that polyphenol non-covalent binding induces tertiary rearrangement and alters the tryptophan microenvironment in whey proteins [41,44].

### 3.5. Synchronous Fluorescence Spectroscopy Analysis

Synchronous fluorescence spectroscopy (SFS) monitors amino acid microenvironments by scanning excitation and emission with a constant wavelength offset. Usually, Δλ values of 15 nm and 60 nm correspond to Tyr and Trp, respectively [45]. From Figure 2c, at Δλ = 15 nm, the WPI-EA non-covalent complex showed reduced fluorescence intensity compared to WPI, but the maximum emission wavelengths remained unchanged (285 nm for 0.05 mg/mL; 295 nm for 0.1 and 0.2 mg/mL), indicating that EA binding did not alter the Tyr microenvironment but still caused fluorescence quenching. In contrast, at Δλ = 60 nm (Figure 2d), the maximum emission peaks of the WPI-EA non-covalent complex (0.05, 0.1, 0.2 mg/mL) underwent red shifts of 0.2, 0.2, and 1.2 nm, respectively, alongside a fluorescence intensity drop. Thus, EA addition altered the WPI microenvironment, changed its secondary structure, increased polarity and decreased hydrophobicity around Trp residues, and loosened the tertiary structure. Notably, the larger red-shift amplitude at 0.2 mg/mL (1.2 nm) compared to lower concentrations (0.2 nm) suggested that the disturbance of the Trp microenvironment by EA was more pronounced at a relatively higher protein concentration. This is consistent with Wang et al. [46], who reported that hesperidin was embedded within the hydrophobic cavity of whey protein, causing the microenvironments of tyrosine and tryptophan residues and leading to fluorescence quenching.

To further explore this, we compared synchronous fluorescence spectra at different wavelengths. From Figure 2c,d, the WPI-EA non-covalent complex fluorescence intensity exhibited a larger reduction at Δλ = 60 nm than at Δλ = 15 nm, suggesting that after non-covalent binding, the synchronous fluorescence is mainly attributable to tryptophan residues, with the EA binding site situated closer to that residue. Moreover, the maximum fluorescence intensities of both tyrosine and tryptophan in the WPI-EA non-covalent complex were lower than those in WPI, suggesting that EA shielded the fluorescence signals of these residues. These spectral findings closely matched those previously described by Hao et al. [44]. In summary, EA binding primarily altered the Trp microenvironment, leading to a more relaxed protein conformation and reducing fluorescence emission of both Tyr and Trp via spatial proximity or energy transfer.

### 3.6. Three-Dimensional Fluorescence Spectroscopy Analysis

**Figure 3:**
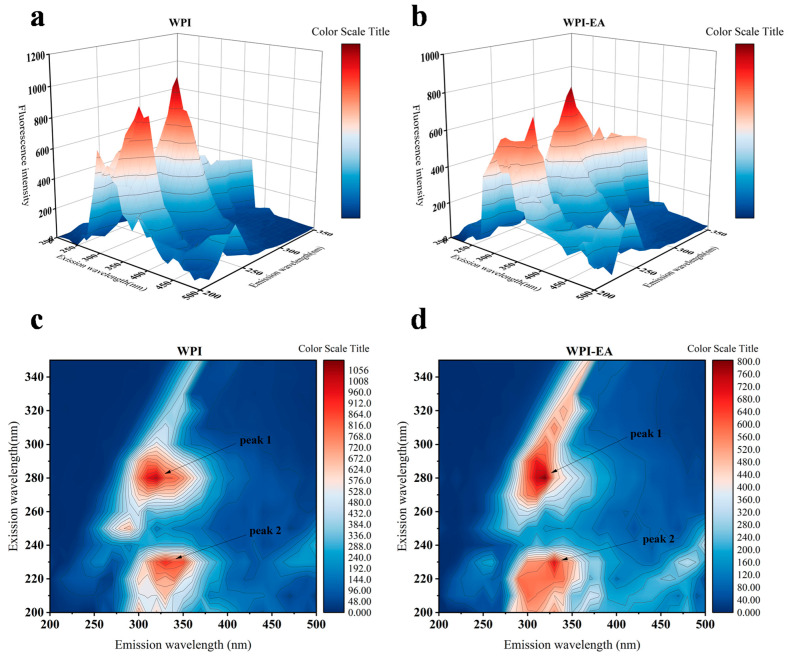
3D fluorescence spectrum of WPI (**a**) and WPI-EA non-covalent complex (**b**); 3D fluorescence contour map of WPI (**c**) and WPI-EA non-covalent complex (**d**).

Three-dimensional fluorescence (3D-FL) spectroscopy can characterize protein conformational changes and ligand-induced alterations in the microenvironment. From Figure 3, both WPI and the WPI-EA non-covalent complex displayed two characteristic peaks. Peak 1 reflected the intrinsic fluorescence of Trp and Tyr residues, while peak 2 was assigned to the protein backbone vibrations [47]. Table 2 shows that for the WPI-EA non-covalent complex, peak 1 intensity decreased by 22.0%, with no blue or red shift observed. The hypochromic effect indicated that the interaction of EA with WPI quenched the fluorescence of aromatic residues [30]. Combined with synchronous fluorescence results, EA bound near Trp residues, increasing local polarity and decreasing hydrophobicity. Peak 2 intensity of the WPI-EA non-covalent complex decreased by 31.8% with no spectral shift, suggesting that the non-covalent interaction between EA and WPI induced extension of the polypeptide chain and exposure of some buried hydrophobic amino acids, thereby leading to conformational changes in the protein. Cao et al. also demonstrated that gallic acid and EGCG caused marked conformational changes in WPI and reduced its surface hydrophobicity by half [48]. This finding corroborates our results. In conclusion, EA interacted with aromatic residues and also influenced the conformational state of the polypeptide backbone.

### 3.7. Surface Hydrophobicity (H_0_) Analysis

**Figure 4:**
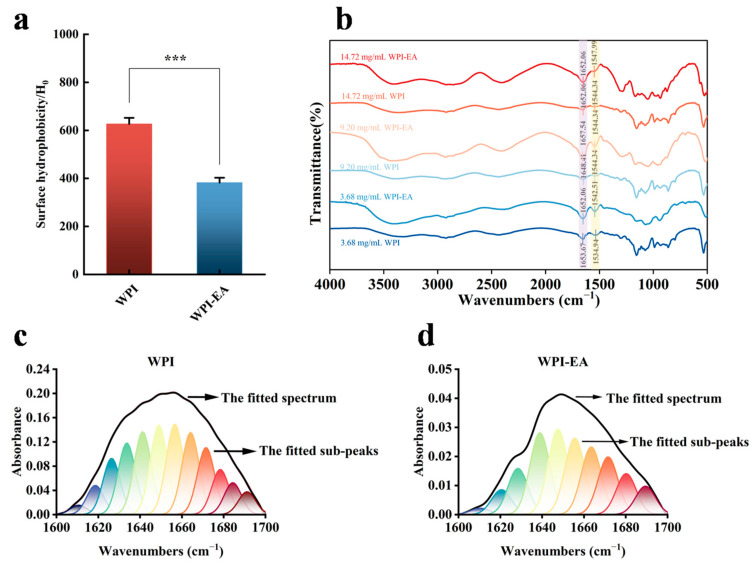
Surface hydrophobicity of WPI and WPI-EA non-covalent complex (**a**); FTIR spectra of 3.68 mg/mL, 9.20 mg/mL, and 14.72 mg/mL WPI and WPI-EA non-covalent complex (**b**); Amide I curve-fitting results for WPI (**c**) and WPI-EA non-covalent complex (**d**). Note: Asterisk (***) indicates statistically significant differences between the WPI and WPI-EA non-covalent complex (*p* < 0.05), *n* = 3.

Hydrophobic interactions played the dominant role in the non-covalent binding between proteins and polyphenols. These interactions primarily occurred at the interface between hydrophobic regions of the protein and aromatic rings of polyphenols, reflecting changes in the tertiary structure of the protein [25]. Figure 4a and Table 3 show that the surface hydrophobicity of the WPI-EA non-covalent complex (382.54 ± 20.27) decreased significantly compared with that of WPI (626.48 ± 25.86). Levene’s test confirmed the homogeneity of variances between the two groups (F = 0.23, *p* = 0.655). Subsequently, the independent samples *t*-test indicated a statistically significant difference (*p* < 0.05). This effect can be mainly attributed to the distribution of multiple phenolic hydroxyl groups from EA on the protein surface following complex formation, which leads to an increase in the number of hydrophilic groups. At the same time, EA occupied hydrophobic sites on WPI that would otherwise bind the ANS probe, reducing the detectable hydrophobic area. Combined with fluorescence results, EA binding induced peptide chain folding and aromatic residue burial, thereby exposing previously hidden hydrophilic groups and additionally decreasing the surface hydrophobicity. Shahidi et al. [49] pointed out that the unique molecular architecture of a polyphenol and the circumstances under which it binds to a protein dictate whether the protein will fold or unfold. Our observations are consistent with the reports by Meng et al. [30] and Hao et al. [50], who found that polyphenols binding to WPI or pea protein isolate significantly reduced surface hydrophobicity, indicating changes in protein conformation and physicochemical properties. Collectively, the decreased surface hydrophobicity of the WPI-EA non-covalent complex confirmed that effective EA binding induced tertiary structural rearrangement and redistribution of hydrophobic regions.

### 3.8. FT-IR Spectroscopy Analysis

From Figure 4b, the amide I bands of WPI at different concentrations (3.68, 9.20, and 14.72 mg/mL) were located at 1653.67, 1648.41, and 1652.06 cm^−1^, respectively, while those of the WPI-EA non-covalent complex shifted to 1652.06, 1657.54, and 1652.06 cm^−1^, respectively. To identify the specific peak positions of the amide I (1600–1700 cm^−1^) and amide II (1500–1600 cm^−1^) bands, we referred to previously reported wavenumber ranges for protein secondary structures [51] and performed peak picking using the peak analysis function in Origin 2022 software. When EA bound non-covalently to WPI, the amide I band exhibited a frequency shift. This shift arose from vibrational coupling effects that occurred when phenolic compounds formed hydrogen bonds with the C=O groups of the peptide backbone [52]. Similarly, the amide II bands of WPI (3.68, 9.20, and 14.72 mg/mL) were at 1534.94, 1544.34, and 1544.34 cm^−1^, while those of the WPI-EA non-covalent complex shifted to 1542.51, 1544.34, and 1547.99 cm^−1^, respectively. The amide II band, which arose mainly from N-H bending and C-N stretching vibrations, exhibited a strong sensitivity to the formation of hydrogen bonds [53]. These amide II band shifts, observed across the tested concentration range, further corroborated that EA and WPI engage in intermolecular hydrogen-bond interactions.

The amide I band shown in Figure 4c,d was subjected to deconvolution and curve fitting with Peakfit, from which the secondary structure compositions of WPI and its EA complex were derived (Table 4). Levene’s test confirmed equal variances between WPI and the WPI-EA non-covalent complex for all four secondary structure components (β-sheet: F = 0.23, *p* = 0.66; random coil: F = 0.014, *p* = 0.912; α-helix: F = 0.053, *p* = 0.829; β-turn: F = 0.54, *p* = 0.503). Subsequent independent samples *t*-tests revealed that the contents of β-sheet, random coil, and α-helix differed significantly between the two samples, whereas β-turn content did not. Detailed statistical results are provided in Table S6. The WPI-EA non-covalent complex showed lower proportions of random coil and β-turn but higher β-sheet and α-helix than native WPI, implying that EA binding drove a conformational change that shifted random coil and β-turn toward β-sheet and α-helix. Maintenance of the α-helical conformation depends largely on internal hydrogen bonding between the C=O and N-H moieties [54]. Therefore, the increased α-helix content in WPI-EA might be attributed to enhanced hydrogen bonding. Furthermore, EA is rich in phenolic hydroxyls, carbonyls, and aromatic rings, which can form hydrogen bonds and other polar interactions with WPI amino acid residues, thereby promoting conformational rearrangement. Zhao et al. found that EA increased the α-helix and β-sheet content of zein while decreasing the random coil content, suggesting that polyphenol structure has a significant impact on protein secondary structure [55]. This observation is consistent with our results. Additionally, the increased α-helix content in the WPI-EA non-covalent complex suggests a looser structure, which may potentially improve solubility and consequently enhance protein bioaccessibility, consistent with Meng et al. [30].

### 3.9. Microstructure Analysis

**Figure 5:**
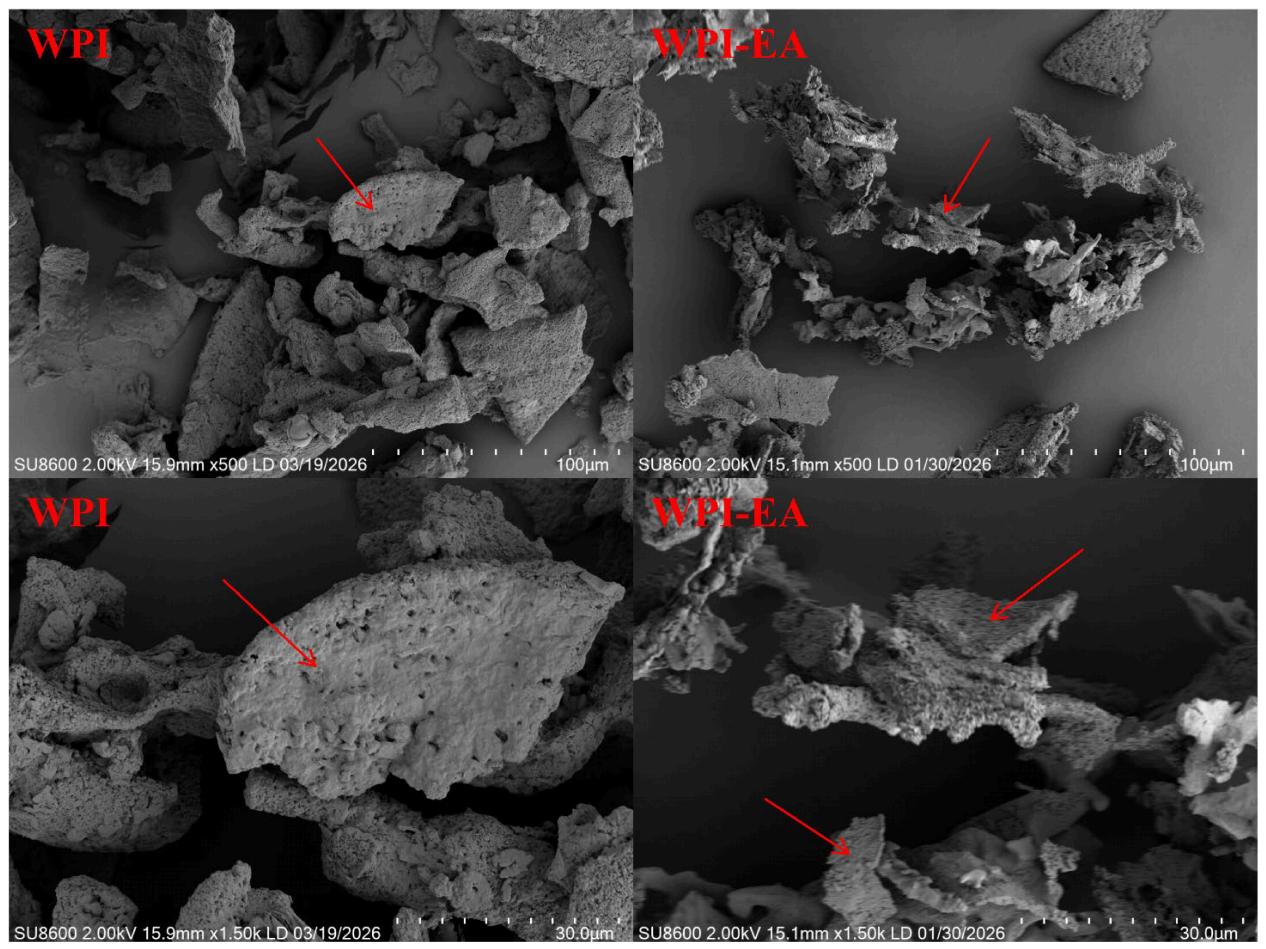
SEM images of WPI and WPI-EA non-covalent complexes at magnifications of 500× and 1500×.

From Figure 5, both WPI and the WPI-EA non-covalent complex exhibited a lamellar microstructure. However, WPI had rough, irregular edges with obvious cavities, while the WPI-EA non-covalent complex showed smoother, more uniform surfaces, clearer edges, and a smaller sheet size. This morphological transition was closely related to protein conformational rearrangement [56]. Combined with the surface hydrophobicity results, EA significantly reduced WPI surface hydrophobicity by burying hydrophobic regions, which transformed the previously aggregated lamellae into ordered arrays [31]. These observations are consistent with the FTIR analysis, which showed that the transition from random coil to β-sheet and α-helix in the WPI-EA non-covalent complex indicates the formation of a more ordered secondary structure framework. This transition concomitantly reduces random chain entanglements, yielding clearer lamellar boundaries and a more uniform size distribution, which is in agreement with the findings of Meng et al. [30]. Furthermore, the ordered conformation enhanced the tolerance of the protein to heating and shearing during food processing, potentially contributing to its bioaccessibility [57].

### 3.10. Molecular Docking Analysis

**Figure 6:**
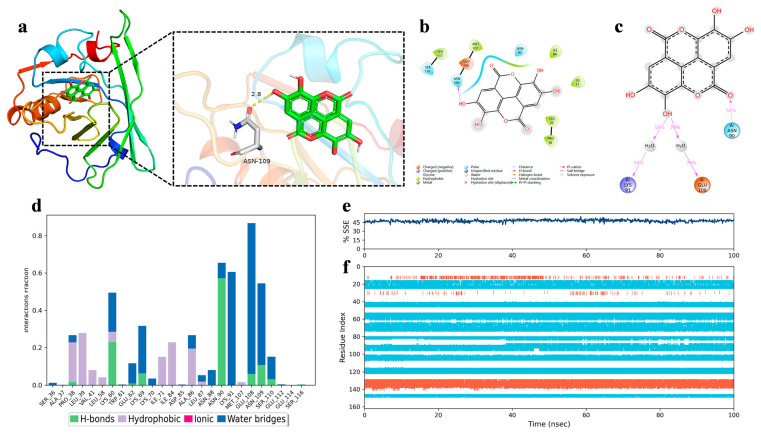
The best molecular docking analysis on the interaction between β-lactoglobulin and EA (**a**) and the 2D interaction diagram (**b**); the yellow dotted line and purple arrow illustrate the hydrogen-bond interaction. Legend of hydrogen bond, hydrophobic, ionic, and water bridge interactions in the β-Lg-EA system (**c**). The interaction ratio of key amino acid residues between EA and β-Lg in β-Lg-EA (**d**); β-Lg-EA protein secondary structure content evolution (**e**). Secondary structure distribution of amino acid residues in β-Lg-EA (**f**).

Given that β-Lg constitutes over 70% of WPI, the β-Lg-EA complex was selected for molecular simulation. Figure 6a shows EA binding to the protein surface with the lowest binding energy of −5.546 kcal/mol, indicating spontaneous and stable binding. The EA-β-Lg interaction pattern was presented in Figure 6b. Five hydrophobic interactions and one hydrogen bond (Asn109) were formed between EA and β-Lg, which together maintained complex stability. The number of hydrophobic interactions significantly outnumbered the number of hydrogen bonds, indicating that hydrophobic forces dominate the binding process. This observation is consistent with the structural features of β-Lg, which contains a hydrophobic β-barrel cavity that can accommodate a variety of nonpolar ligands. In the case of EA, hydrophobic contacts likely involve its polycyclic aromatic backbone interacting with nonpolar side chains of residues, including LEU39, PRO38, ALA86, ILE84, and ILE71, while the hydrogen bond with ASN109 originates from the phenolic hydroxyl groups. This binding mode, in which hydrophobic interactions act as the main driving force while hydrogen bonds provide directional specificity, is consistent with recent reports on β-Lg interactions with other polyphenols, including resveratrol, chlorogenic acid, and olive biophenolic secoiridoids [58,59]. This observation was strongly supported by both the surface hydrophobicity assay, which showed reduced hydrophobicity of the WPI-EA non-covalent complex, and the synchronous fluorescence results, which indicated a rearrangement of hydrophobic regions and the environments of aromatic residues. Although EA is bound mainly on the surface of β-Lg, the embedding of hydrophobic groups and hydrogen-bond formation likely induced conformational adjustments in the backbone, exposing Trp and Tyr to a more polar microenvironment [60]. The observed fluorescence quenching and wavelength shifts can thus be explained at the molecular level by these conformational rearrangements. Based on the optimal docking conformation, we then performed molecular dynamics simulations to verify the stability of the complex.

### 3.11. Molecular Dynamics Simulation Analysis

**Figure 7:**
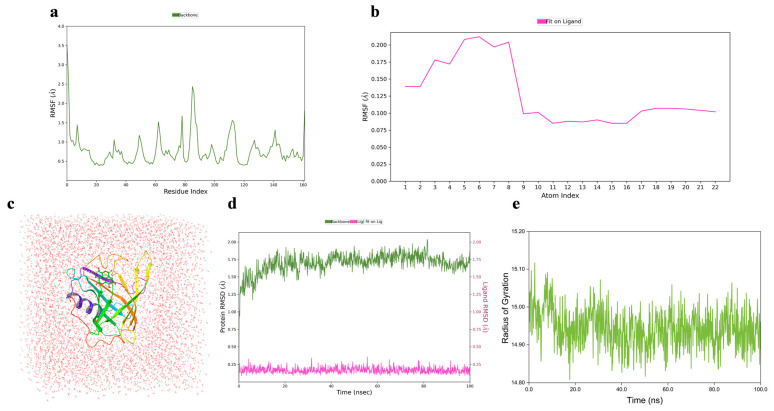
The RMSF (**a**,**b**), RMSD (**d**), and Rg (**e**) for β-lactoglobulin in the absence and presence of EA; Movement path of EA in the binding site during 100 ns (**c**).

To further investigate the interaction mechanism, we chose the most favorable EA-β-Lg conformation for MD simulations. Molecular dynamics simulations were performed in triplicate using independently generated initial velocities to ensure reproducibility. Trajectory convergence was assessed by monitoring RMSD stabilization over time; all simulations reached equilibrium after approximately 80 ns, and the final 20 ns were used for analysis. The root-mean-square deviation (RMSD) is a standard metric used to assess both the equilibration of a simulation system and the structural integrity of a protein. Higher RMSD values indicate greater conformational instability. When the RMSD fluctuation averaged over the trajectory fell below 0.1 nm, the system was deemed to have reached a dynamically stable state [61]. According to Figure 7d, following roughly 100 ns of MD, the mean RMSD for β-Lg and β-Lg-EA reached 2 Å and 0.25 Å, respectively. The RMSD of the ligand was calculated as “Lig fit Lig” in the Desmond output, which reflects internal atomic fluctuations relative to the initial conformation of the ligand, rather than displacement relative to the protein binding pocket. Given that EA is a relatively rigid molecule with multiple aromatic rings and lactone groups, the observed value of 0.25 Å is reasonable and suggests that the ligand maintained a stable internal conformation throughout the simulation. The lower RMSD of β-Lg-EA indicated that the binding of EA to β-Lg involved hydrogen bonds and other forces, reducing the conformational flexibility of β-Lg and enhancing the stability of EA. Furthermore, the minor fluctuations of both protein and ligand imply a very stable β-Lg-EA complex. The RMSD value and movement of EA (Figure 7c) also confirmed that EA remained stably bound to the surface of β-Lg.

The root-mean-square fluctuation (RMSF) provided a measure of both residue conformational freedom and the local dynamic behavior of secondary structure during the simulation [62]. Lower RMSF values generally indicate higher structural rigidity and stability. Figure 7a shows that the RMSF values of the protein backbone remained below 2.5 Å for most residues throughout the simulation, indicating that the overall structure of β-Lg was well maintained upon EA binding. Similarly, the ligand RMSF values remained below 0.2 Å (Figure 7b), reflecting the internal stability of EA within the binding site. The reduced fluctuations can be attributed to the formation of hydrogen bonds between the phenolic hydroxyl groups of EA and nearby amino acid residues, which constrain local backbone flexibility and limit the mobility of the ligand. This observation aligns with previous MD studies on polyphenol-protein systems, which demonstrated that hydrogen-bonding networks decrease RMSF values and stabilize the bound conformation of ligands [60]. The low RMSF values thus confirm that EA maintained a stable binding pose throughout the simulation without undergoing major conformational rearrangement.

The radius of gyration (Rg) is an indicator of system compactness, with higher Rg values reflecting greater molecular expansion and reduced binding stability [63]. Figure 7e shows that the average Rg of the β-Lg-EA complex gradually decreased and remained relatively stable throughout the 100 ns molecular dynamics simulation, indicating the formation of a stable complex. This reduction in Rg can be ascribed to two cooperative factors: the insertion of the polycyclic aromatic skeleton of EA into the hydrophobic pocket of β-Lg, which promotes hydrophobic packing, and the establishment of hydrogen bonds between the phenolic hydroxyl groups of EA and neighboring residues, leading to a more compact polypeptide arrangement around the binding site [64]. These findings are consistent with the scanning electron microscopy results and the observations of Janbaz-Amirani et al. [60], who also reported that ligand-induced conformational compaction contributes to complex stability.

The formation and stability of protein–ligand hydrogen bonds are key parameters for assessing the conformational stability of complexes. After around 100 ns of simulation, β-Lg-EA formed three stable hydrogen bonds and many hydrophobic interactions (Figure 6c,d), including a direct hydrogen bond with ASN90 and water-mediated hydrogen bonds with LYS91 and GLU108, along with multiple hydrophobic interactions involving residues such as LYS60, LEU39, PRO38, ALA86, ILE84, and ILE71. The coexistence of hydrogen bonds and hydrophobic interactions throughout the simulation confirms that both forces contribute to stability, with hydrophobic interactions as the primary driving force and hydrogen bonds as auxiliary, consistent with Janbaz-Amirani et al. [60]. Chen et al. found that ASN and LYS in water-mediated H-bond networks stabilize WPI-EGCG complexes [65].

Protein secondary structure is a key indicator of stability and functionality. Its evolution revealed ligand-induced conformational changes. After a 100 ns MD simulation, the effect of EA on β-Lg secondary structure was analyzed (Figure 6e,f). Throughout the simulation, β-sheet and α-helix contents remained stable, with no unfolding or conformational jumps. The nearly unchanged secondary structure assignment for each residue suggests that the β-Lg polypeptide backbone was not significantly affected by EA. Thus, the stability of the β-Lg-EA complex mainly arose from tertiary structure adjustments rather than major secondary structure rearrangements. Similarly, Ma et al. found that WPI binding to galangin or genistein induced only minor secondary changes, with major tertiary-level adaptations [25].

### 3.12. Combining Free Energy Analysis and Energy Decomposition

Combining free energy reflected protein–ligand affinity. The MM-GBSA calculation gave −37.51 kcal/mol for β-Lg-EA. The negative sign indicated a spontaneous binding process and a high degree of stability of the resulting complex [60]. This value is consistent with reported polyphenol-protein complexes, confirming strong interactions [66]. The observed high binding affinity likely arose from the cooperative interplay between H-bonding networks and hydrophobic contacts. Thus, the combined binding free energy, hydrogen-bond analysis, and secondary-structure stability collectively confirmed stable noncovalent binding between EA and WPI and provided a structural basis for future anti-sarcopenia studies.

This study first identified potential EA-β-Lg binding sites via molecular docking, then used MD simulations to analyze the binding mode, free energy, and conformational dynamics. Due to differences between theoretical and actual solution structures, combining molecular docking, MD, and UV-Vis spectroscopy enabled more accurate elucidation of the binding mechanism [65]. Zhang et al. also found that molecular simulation results corroborated *in vitro* release experiments when studying the effects of ultrasonication and homogenization on EGCG encapsulation in WPI-pectin particles [67]. However, objective differences still exist between theoretical models and complex food systems. In addition, we acknowledge that the formation of the WPI-EA non-covalent complex was primarily inferred from spectroscopic measurements, computational analyses, and indirect functional evidence, rather than from direct biophysical measurements such as isothermal titration calorimetry. This limitation will be addressed in future studies by incorporating complementary thermodynamic and binding assays.

## 4. Conclusions

In this study, we optimized the preparation of WPI-EA non-covalent complexes via single-factor and orthogonal experiments and explored the interaction mechanism using multi-spectroscopic techniques combined with molecular simulations. The optimal conditions were pH 5.0, a reaction time of 2 h, and a WPI-to-EA molar ratio of 1:2.5. Under these conditions, the complex showed markedly enhanced antioxidant activity while maintaining good digestibility. Although total amino acid and cysteine contents decreased, the branched-chain and essential amino acid levels remained comparable to those of WPI, which is a favorable feature for muscle protein synthesis. Spectroscopic analyses indicated that EA binding modified the secondary and tertiary structures of WPI, as reflected by reduced hydrophobicity around tyrosine and tryptophan residues, an extended peptide backbone, increased α-helix content, a looser overall conformation, and a smoother, more uniform morphology. Molecular docking and dynamics simulations revealed that hydrophobic interactions were the primary driving force for EA binding to β-Lg, with hydrogen bonds contributing additional stability. The calculated binding free energy further confirmed the spontaneous and stable nature of the association. These conformational changes provide a mechanistic basis for the improved antioxidant and structural properties of the complex. Admittedly, the current work is limited to physicochemical characterization and *in vitro* digestion assessment; the potential applicability of the complex in sarcopenia-related contexts requires validation through cellular and animal experiments, which will form the core of our subsequent research.

## Figures and Tables

**Table 1:** Amino acid content in the supernatant after *in vitro* digestion of the WPI-EA non-covalent complex.

Amino Acid	WPI (g/100 g)	WPI-EA (g/100 g)
Asp	1.29	1.06
Thr	0.83	0.69
Ser	0.54	0.52
Glu	2.07	1.66
Pro	0.65	0.50
Gly	0.22	0.20
Ala	0.60	0.50
Cys	0.63	0.22
Val	0.74	0.48
Met	0.06	0.03
Ile	0.81	0.65
Leu	1.25	0.99
Tyr	0.61	0.63
Phe	0.46	0.41
Lys	0.90	0.72
His	0.21	0.18
Arg	0.34	0.29
Total	12.19	9.74

**Table 2:** 3D fluorescence spectrum results on the tertiary structures of WPI and WPI-EA non-covalent complex.

		Peak 1 (The Shorter One)			Peak 2 (The Taller One)	
	peak	intensity	change	peak	intensity	change
	λ_ex_/λ_em_	F_0_	%	λ_ex_/λ_em_	F_0_	%
WPI	280/320	1028.4		230/330	912.0	
WPI-EA	280/320	802.6	22.0	230/330	622.2	31.8

**Table 3:** Surface hydrophobicity (H_0_) of WPI and WPI-EA non-covalent complex.

	H_0_ Value	Change (%)	t Value	*p* Value
WPI	626.48 ± 25.86		12.86	<0.001
WPI-EA	382.54 ± 20.27 ***	38.94		

Note: Asterisk (***) indicates statistically significant differences between the WPI and WPI-EA non-covalent complex (*p* < 0.05), *n* = 3.

**Table 4:** The secondary structure contents of WPI and WPI-EA non-covalent complex samples.

Name	β-Sheet (%)	Random Coil (%)	α-Helix (%)	β-Turn (%)
WPI	24.56 ± 0.46	25.40 ± 0.47	13.32 ± 0.31	37.99 ± 0.32
WPI-EA	30.85 ± 0.62 *	16.55 ± 0.43 *	15.68 ± 0.37 *	37.78 ± 0.18

Note: Asterisk (*) indicates statistically significant differences between the WPI and WPI-EA non-covalent complex (*p* < 0.05), *n* = 3.

## Data Availability

The original contributions presented in this study are included in the article/Supplementary Materials. Further inquiries can be directed to the corresponding authors.

## Supplementary Materials

The following supporting information can be downloaded at https://www.mdpi.com/article/10.3390/foods15152689/s1; Table S1: The factors of the orthogonal test on the WPI-EA non-covalent complex. Table S2: Orthogonal test and range analysis of the WPI-EA non-covalent complex. Table S3: Analysis of variance in the WPI-EA non-covalent complex. Table S4: Analysis of variance for the DPPH radical scavenging activity in single-factor experiments. Table S5: Analysis of variance for the protein digestibility in single-factor experiments. Table S6: Independent sample *t*-test analysis of secondary structure content between WPI and WPI-EA.

## Abbreviations

WPI	Whey protein isolate
EA	Ellagic acid
DMSO	Dimethylsulfoxide
SGF	Simulated gastric fluid
SIF	Simulated intestinal fluid
DPPH	1,1-diphenyl-2-picrylhydrazyl
ANS	8-Anilino-1-naphthalenesulfonic acid
UV-vis	Ultraviolet (UV)–visible
SFS	Synchronous fluorescence spectroscopy
3D-FL	Three-dimensional fluorescence
SEM	Scanning electron microscope
β-Lg	β-lactoglobulin
MD	Molecular dynamics
RMSD	Root-mean-square deviation
RMSF	Root-mean-square fluctuation
Rg	Radius of gyration
MM-GBSA	Molecular mechanics–generalized born surface area
