# Supplementary material for: Morphological Characterization, Interaction Mechanisms, and Functional Properties of a Non-Covalent Whey Protein Isolate–Ellagic Acid Complex
Source: Foods. 2026 Jul 30;15(15):2689. doi: 10.3390/foods15152689 (PMC13465059; doi:10.3390/foods15152689)
Supplement: Supplementary file 1 [file foods-15-02689-s001.zip › foods-4418687-supplementary.pdf]

Table S1 The factors of the orthogonal test on the WPI-EA non-covalent complex

Levels	Factors			
	A	B	C	D
	pH	Blank	Reaction time (h)	Molar concentration ratio of reactants (WPI_{mol}: EA_{mol})
1	5.0	0	1.5	1:1.5
2	5.5	0	2.0	1:2.0
3	6.0	0	2.5	1:2.5

Table S2 Orthogonal test and range analysis of the WPI-EA non-covalent complex

Test number	pH	Blank	Reaction time (h)	Molar concentration ratio of reactants (WPImol: Eamol)	DPPH Free Radical Scavenging (%)	Protein digestibility (%)	DPPH clearance affiliation	Protein digestibility affiliation	Comprehensive score
1	1	1	1	1	44.21±0.47	84.94±0.82	0.23	0.88	0.49
2	1	2	2	2	49.41±0.71	75.98±1.33	0.80	0.53	0.69
3	1	3	3	3	45.63±0.85	86.50±1.11	0.39	0.94	0.61
4	2	1	2	3	51.22±0.90	86.71±0.54	1.00	0.95	0.98
5	2	2	3	1	49.88±0.24	65.75±2.24	0.85	0.13	0.56
6	2	3	1	2	42.79±0.47	88.04±1.66	0.08	1.00	0.45
7	3	1	3	2	50.12±0.63	62.51±5.18	0.88	0.01	0.53
8	3	2	1	3	42.08±0.63	84.06±0.20	0.00	0.85	0.34
9	3	3	2	1	50.75±0.14	62.36±2.69	0.95	0.00	0.57
K1	1.30	2.00	0.78	1.13					
K2	1.01	1.60	1.55	0.98					
K3	0.91	1.62	1.09	1.32					
k1	0.43	0.67	0.26	0.38					
k2	0.34	0.53	0.52	0.33					
k3	0.30	0.54	0.36	0.44					
Range	0.13	0.13	0.25	0.11					
The relative importance of factors	Reaction time > pH > Molar concentration ratio of reactants								
The optimal preparation conditions	2 h, pH 5.0, 1:2.5								

Table S3 Analysis of variance in the WPI-EA non-covalent complex

Source of Variation	Sum of Squared Deviations	Degree of Freedom	Mean square	F value
pH	0.05	2.00	0.03	1.79
Reaction time	0.15	2.00	0.08	5.04
Molar concentration ratio of reactants	0.03	2.00	0.01	0.88

Table S4 Analysis of variance for the DPPH radical scavenging activity in single-factor experiments

Factors	Sum of Squared Deviations	Degree of Freedom	Mean square	F value	p value
pH	172.58	4.00	43.14	9.54	0.002
Reaction time	141.78	4.00	35.44	6.20	0.009
Molar concentration ratio of reactants	1238.61	4.00	309.65	32.02	<0.001

Table S5 Analysis of variance for the protein digestibility in single-factor experiments

Factors	Sum of Squared Deviations	Degree of Freedom	Mean square	F value	p value
pH	1702.13	4.00	425.53	23.24	<0.001
Reaction time	73.09	4.00	18.27	5.81	0.011
Molar concentration ratio of reactants	531.11	4.00	132.78	11.67	0.001

Table S6 Independent sample t-test analysis of secondary structure content between WPI and WPI-EA

		Levene's Test		t-test		
		F value	p value	t value	Degree of Freedom	Sig (2-tailed)
β-sheet	equal variances assumed	0.23	0.657	-14.14	4.00	<0.001
	equal variances not assumed			-14.14	3.67	<0.001
random coil	equal variances assumed	0.01	0.912	24.18	4.00	<0.001
	equal variances not assumed			24.18	3.98	<0.001
α-helix	equal variances assumed	0.05	0.829	-8.59	4.00	0.001
	equal variances not assumed			-8.59	3.88	0.001
β-turn	equal variances assumed	0.54	0.503	1.02	4.00	0.364
	equal variances not assumed			1.02	3.15	0.379
